# Simulated microgravity triggers a membrane adaptation to stress in *E. coli* REL606

**DOI:** 10.1186/s12866-025-04064-7

**Published:** 2025-06-09

**Authors:** Brittney Lozzi, Lea Adepoju, Josh L. Espinoza, Michael Padgen, Macarena Parra, Antonio Ricco, Sarah Castro-Wallace, Jeffrey E. Barrick, Aubrie O’Rourke

**Affiliations:** 1 Space Center Office of STEM Engagement (OSTEM) Intern Program, NASA Kennedy, Kennedy Space Center, Merritt Island, FL 32899 USA; 2 Program in Genetics and Genomics, Baylor College of Medicine, Houston, TX 77030 USA; 3 Earth and Atmospheric Sciences, Georgia Institute of Technology, Atlanta, GA 30332 USA; 4 Department of Environment and Sustainability, J. Craig Venter Institute, 4120 Capricorn Way, San Diego, CA 92037 USA; 5 NASA Ames Research Center, Moffett Field, CA USA; 6 Biomedical Research and Environmental Sciences Division, NASA Johnson Space Center, Houston, TX USA; 7 Department of Molecular Biosciences, Center for Systems and Synthetic Biology, The University of Texas at Austin, 2500 Speedway A5000, Austin, TX 78751 USA; 8 NASA Exploration Research and Technology, Kennedy Space Center, Merritt Island, FL 32899 USA

**Keywords:** Simulated microgravity, E. coli, REL606, Gene expression, Experimental evolution

## Abstract

Investigating the evolution of *Escherichia coli* in microgravity offers valuable insights into microbial adaptation to extreme environments. Here the effects of simulated microgravity (SµG) on gene expression and genome evolution of *E. coli* REL606, a strain evolved terrestrially for 35 years, is explored. The transcriptomic changes for glucose-limited and glucose-replete conditions over 24 h illustrate that SµG increased the expression of genes involved in stress response, biofilm, and metabolism. A greater number of differentially expressed genes related to the general stress response (GSR) and biofilm formation is observed in simulated microgravity cultures under glucose-limited conditions in comparison to glucose-replete conditions. Longer term SµG culture under glucose-limited conditions led to the accumulation of unique mutations when compared to control cultures, particularly in the *mraZ/fruR* intergenic region and the *elyC gene*, suggesting changes in peptidoglycan and enterobacterial common antigen (ECA) production. These findings highlight the physiological and genomic adaptations of *E. coli* to microgravity, offering a foundation for future research into the long-term effects of space conditions on bacterial evolution.

**Supplementary Information:**

The online version contains supplementary material available at 10.1186/s12866-025-04064-7.

## Background

Understanding how microgravity influences the behavior and physiology of microorganisms is crucial for ensuring the success and safety of long-duration space missions. The selective pressures in the spaceflight environment are combinatorial and among these are radiation, microgravity, and reduced hydrostatic pressure. Experiments conducted in space and simulated microgravity (SµG) on Earth have demonstrated that various aspects of bacterial behavior and physiology, including bacterial morphology, growth, biofilm formation, metabolism, gene transfer, antibiotic resistance, and pathogenicity can be altered in *Escherichia coli* [1–5], *Bacillus subtilis* [6], *Salmonella enterica* [7–11], *Staphylococcus aureus* [12], *Pseudomonas aeruginosa* [13], *Ralstonia pickettii* [14], and *Burkholderia contaminans* [15]. Studies have also demonstrated that these outcomes can be modulated by the availability of nutrients such as phosphate and organic carbon [16]. Prior spaceflight experiments spanning 36–49 h with non-motile *E. coli* have demonstrated a shortened lag phase and an extended exponential growth phase, with flight samples reaching a saturated cell population almost double that of ground controls [17]. However, other experiments reported no difference in growth parameters for another non-motile strain of *E. coli* in spaceflight [18]. The first account of increased growth is from Klaus et al*.* 1997 that reported on the datasets from seven Space Shuttle experiments with asynchronous ground controls [17]. Follow-on work from Kacena et al*.* 1999 reported on three time points collected from two biological replicates throughout the growth curve to determine the significance of the flight experiment as compared to synchronous ground controls [19]. The authors concluded that the observed growth effect is the result of decreased mass transfer. It was further hypothesized that the absence of gravity-driven cell sedimentation by convection directly affected the environment around the bacteria and led to the accumulation of acid, quorum-sensing molecules, and waste products, which altered cell growth [17]. No difference in growth was observed when cells were grown on agar substrate. Therefore, the indirect effects of microgravity on bacteria are more phenotypically evident in the liquid environment than for an agar substrate after short-term culture [20].

Long-duration spaceflight is expected to generate sustained genomic and phenotypic changes in bacteria ultimately shaping the organism to exhibit the best-suited “space traits” for survival. To date, no continuous culture microbial monitoring experiment has been reported for spaceflight. The most relevant data exists from the surface, air, and water monitoring efforts used in microbial surveillance of the International Space Station (ISS) as a built environment [21–23]. Here, researchers utilized genomic and metagenomic sequencing to understand how populations change over time. JAXA’s Microbe I, II, and III longitudinal studies illustrated that the population density of the *Enterobacteriaceae* family increased in samples over time [24] while NASA’s Microbial Tracking (MT)-1 and MT-2 showed that the ISS surface microbiome was dominated by organisms associated with human skin [24]. Metagenomic analysis of these samples revealed that pathways involved in antibiotic resistance with cell membrane, DNA gyrase, and protein synthesis modes of action (MOAs) were enriched [24]. Importantly, these monitoring efforts are not controlled experiments, and therefore the genetic make-up of the founding population is not known. As a result, one cannot track the evolution of these populations over time. Future work can fill in this gap to provide a fundamental understanding of the genetic selection imposed on a model organism due to the controlled, well-defined long-term culture in the spaceflight environment. This would be the first of many steps in the planning of a robust bioregenerative life support system or predicting the microbial trajectory of a newly commissioned spacecraft.

A foundational example of terrestrially tracking the evolution of a microbe is the long-term evolution experiment (LTEE) with *E. coli,* which started in the lab of Richard Lenski [25]. The experiment has 12 replicate populations, half of which were founded by each of two strains that are nearly genetically identical: REL606 (cannot metabolize arabinose) and REL607 (revertant of REL606 that can metabolize arabinose). Both strains are non-motile and have no plasmids. The experiment was started with the goal of observing the evolutionary processes of mutation, genetic drift, and natural selection. Each day, 1% of each culture is diluted into fresh glucose minimal media (DM25). Periodically, samples have been frozen and stored, providing an archive of each population at that time point that could at any point later be revived for further experimentation. Today, the LTEE has run for more than 75 K generations [26]. Rapid increases in fitness occurred early on as the populations adapted to new conditions. Over time, fitness gains gradually slowed but did not stop in most populations as they became better adapted to this environment [27]. One exception to this trend was a sudden gain in fitness when one population evolved the ability to metabolize an additional nutrient, citrate, at around 31.5 K generations [28].

**Fig. 1:**
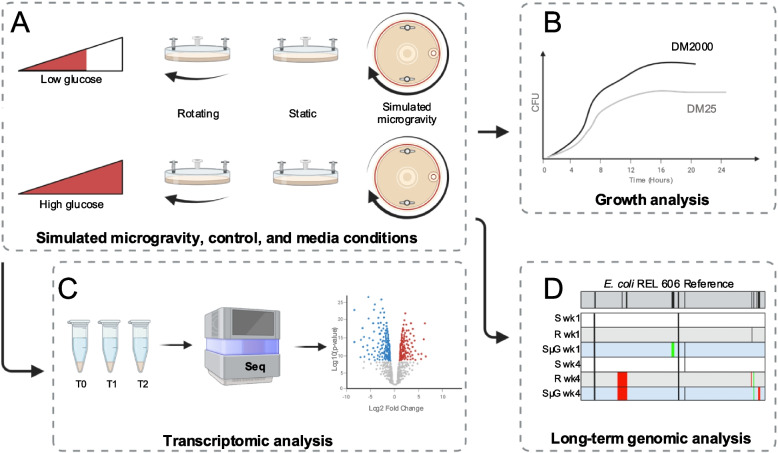
Schematic of project design. **A** *E. coli* REL606 strains were grown in High Aspect Ratio Vessels (HARV) on Rotating Wall Vessels (RWV) for simulated microgravity (SµG) and rotating (R) control in addition to immobile static (S) control. Each growth condition was assayed in glucose-limited (DM25) and glucose-replete (DM2000) media. **B** In a short-term culture study, the growth analysis of SµG, R, and S cultures in both media types was conducted. **C** Also in this short-term study, samples were collected at time of inoculation (T0), at 4.5 h post inoculation in DM25 and 6.5 h post inoculation in DM2000 (T1), and 24 h post inoculation (T2) for each condition for RNAseq and differential gene expression analysis. **D** In a follow-on long-term culture study, samples were passaged each day for 28 days. Samples from every 7th day of culture were subjected to whole genome sequencing to assay the genomic alterations for each growth condition. Created with BioRender.com

We envision the use of the model strain, REL606, as a biosensor for spaceflight effects on the genome given its established history in microbial evolution research in a defined media. To date, one account of a long-term simulated microgravity experiment exists for an *E. coli* strain cultured for 1000 generations, but this work was performed without replication and without a gravity control [29]. The work in this paper serves as a comprehensive exploration of the effects of SμG on *E. coli* REL606, the same strain which founded the LTEE. Here a synthesis of prior spaceflight observations, gene expression, and long-term simulated microgravity experimentation with replication (Fig. 1A-D) is presented. As we embark on a new era of space exploration, understanding how microbes adapt to microgravity is paramount for ensuring the success, safety, and sustainability of future missions. *E. coli* is a key organism in molecular biology and biotechnology as well as a food-borne pathogen making it essential to understand its behavior in closed spaceflight environments.

## Methods

### Bacterial culture

From a freezer stock of REL606, a single colony was obtained by plating on a tetrazolium agar (TA) plate. The colony was inoculated into 3 mL DM2000 media and incubated overnight at 37 °C to obtain an OD600 of 1. From this, 150 uL aliquots were generated and frozen without glycerol to serve as the starting inoculum for the short-term simulated microgravity experiments performed over four weeks. Two 150 µL aliquots of REL606 were removed from the freezer, pelleted, and washed with DM25 and then were each inoculated into 30 mL of DM25. Cells were allowed to incubate with shaking at 37 °C overnight to reach an OD600 of 0.07 and a cell target of 3 × 10^7^ CFU/mL. One 30 mL culture of REL606 was then inoculated into 600 mL of DM25 and the second 30 mL flask of REL606 was inoculated into 600 mL of DM2000. Six 50 mL High Aspect Ratio Vessels (HARVs) were prepared for glucose-limited (DM25: 25 mg/L glucose) experiments and another six 50 mL HARVs were prepared for glucose-replete (DM2000: 2000 mg/L glucose) experiments. Two DM25 HARVs and two DM2000 (Table 1) filled HARVs were loaded on the rotating wall vessel (RWV) vertically for the simulated microgravity (SµG) condition. Two DM25 HARVs and two DM2000 filled HARVs were loaded on the RWV horizontally for the rotating (R) control condition. Two DM25 HARVs and two DM2000 filled HARVs were placed in the incubator horizontally for the static (S) control condition. The experiment was performed in a Percival chamber set to 25 °C and 95% relative humidity. HARVs were rotated at 20 rpm [30], with no forced air from the RWV. The remaining 300 mL of the above 600 mL inoculum for DM25 and DM2000 were sampled as T0 samples and concentrated using the CP SELECT (0.20 µm) and eluted in 250 µl 3 times with 0.075% Tween 20/Tris buffer (InnovaPrep, MO). Cells were further pelleted, the supernatant removed, and then immediately frozen on dry ice and placed in the -80 °C freezer until further processing.

**Table 1:** Davis Minimal media recipes used in this study

	DM25	DM2000
Davis Minimal Media Base Mix	10.6 g/L	10.6 g/L
(ammonium sulfate)	(1.0 g/L)	(7.0 g/L)
(dipotassium phosphate)	(7.0 g/L)	(7.0 g/L)
(magnesium sulfate)	(0.1 g/L)	(0.1 g/L)
(monopotassium phosphate)	(2.0 g/L)	(2.0 g/L)
(sodium citrate)	(0.5 g/L)	(0.5 g/L)
Glucose (10%)	250uL	20 mL
Thiamine (vitamin B1, 0.2%)	1 mL	1 mL

### RNA-sequencing sample preparation

For all short-term culture samples, 20 µL of T0 inoculum was added to 180 µL of DM25 in triplicate and a serial dilution was obtained and plated on TA and then incubated overnight at 37 °C to obtain a CFU/mL count for T0. The same CFU/mL count procedure was done for subsequent timepoints, T1 and T2 for all samples. One HARV from each of the DM25 SµG, R and S conditions was sampled as T1 at 4.5 h. One HARV from each of the DM2000 SµG, R and S condition was sampled as T1 at 6.5 h. The remaining HARVs for DM25 and DM2000 were collected for T2 at 24 h. At each collection event, 50 mL of culture from each of the HARVs was emptied into a 50 mL Falcon tube and then concentrated using the same CP SELECT device as above. Cells were further pelleted, the supernatant removed, and then immediately frozen on dry ice and placed in the -80 °C freezer until further processing. The entire procedure including preculture, RWV culture, and sample collection at T0, T1 and T2 was repeated four times to obtain BR1, BR2, BR3 and BR4. In total, 64 samples (Table 2) were sent on dry ice to Azenta GeneWiz for RNA extraction, ribosomal depletion, library preparation and 2 × 150 base pair, paired-end sequencing. Reads were obtained for each library in an amount of 2 million or greater.

**Table 2:** RNAseq sample counts for the short-term simulated microgravity experiment

	BR 1	BR 2	BR 3	BR4	Media
DM25 (T0)	2	2	2	2	DM25
DM25 R (T1,T2)	2	2	2	2	DM25
DM25 S (T1, T2)	2	2	2	2	DM25
DM25 SuG (T1, T2)	2	2	2	2	DM25
DM2000 (T0)	2	2	2	2	DM2000
DM2000 R (T1,T2)	2	2	2	2	DM2000
DM2000 S (T1, T2)	2	2	2	2	DM2000
DM2000 SuG (T1, T2)	2	2	2	2	DM2000
Sample total	16	16	16	16	64

### RNA-sequencing analysis

The NASA GeneLab RNAseq pipeline was utilized to perform a STAR alignment of reads to the REL606 (NCBI Reference Sequence: NC_012967.1) genome for transcriptomic analysis. Feature counts (v2.0.6) was used to generate a counts table of reads per gene for each sample. Genes with an average read count of 10 reads or less across all samples were removed prior to normalization. DESeq2 (v1.40.2) was used for read count normalization and differential gene expression (DEG) analysis. The DEGs with non-adjusted *p*-values of *p* < 0.05 from each comparison (four comparisons in total) were concatenated to form one list which contained 105 genes. The normalized expression for each of the 105 genes was log2 transformed and Euclidean distances were computed and visualized through hierarchical clustering using pheatmap (version 1.0.12). Gene Ontologies were identified by creating a REL606 database using NCBI Reference Sequence: NC_012967.1 and paring them to the strain MG1655 [31]. The results were visualized using AnnotationHub (v3.8.0), enrichplot (v1.20.3), and clusterProfiler (v4.10.1) with the org.EcK12.eg.db (v3.18.0) database in RStudio. To identify simulated microgravity specific genes, we compared SμG samples in DM25 media to R control samples at T1 and T2. Multiple statistical testing was performed to adjust the *p*-value using the Benjamini–Hochberg method, and differentially expressed genes (DEGs) were identified using log2Fold Change (FC) > 1.25 and adjusted *p*-value < 0.05 cut offs. This process was repeated for DM2000 samples at both timepoints. We determined rpoS enrichment using hypergeometric testing for over-representation of rpoS regulon members with a Benjamini–Hochberg adjusted *p*-value.

### Evolution experiments bacterial culture

From a freezer stock of REL606, four independent colonies (A, B, C, D) were obtained by plating on a TA plate. Each colony was inoculated into 3 mL of DM25 media and incubated overnight at 37 °C to obtain an OD600 of 0.07. A 500 µL aliquot from each of the four DM25 starter cultures (A, B, C, D) was inoculated into 10 mL of DM25 and then loaded into a 10 mL HARV resulting in four HARVs (Biological replicate (BR) A, BR B, BR C or BR D) for each of the three conditions, SµG, R or S. Each culture was allowed to grow for 24 h in the HARVs on a RWV within a Percival chamber set to 25 °C and 95% relative humidity. HARVs were rotated at 20 rpm, with no forced air from the RWV.

A second set of twelve 10 mL autoclavable HARVs were prepared, where 100 µL of the 24-h culture was diluted into 9.9 mL of fresh DM25 media then loaded into the fresh HARV. The newly prepared HARV was then reattached to the RWV in the orientation respective to the designated treatment. HARVs containing the remaining 9.9 mL of spent media from the prior 24-h growth period were drained, the spent media culture was bleach and discarded, and caps and plugs were removed. Empty HARVs were rinsed in Micro® soap diluted 1:20, screws were loosened, HARVs were rinsed in DI water, and soaked overnight. Caps, plugs, and HARVs were wrapped in aluminum foil and autoclaved for 20 min at 121 °C. HARVs were allowed to cool, then screws were tightened, and caps and plugs were reassembled in a sterile hood. Sterile syringes were used to load HARVs with culture. Culture transfer and HARV and media preparation were performed daily for 28 days.

### Whole genome sequencing and mutation calling

For each HARV, a sample was collected every 7th day and plated on TA agar to ensure REL606 sample purity as the sample turns red on this selective agar due to the inability to metabolize arabinose. From the weekly sampling, one -80 °C freezer stock was made with 15% glycerol and another without. CFU/mL counts were obtained to ensure a count of 10^7^ CFU/mL was not exceeded. One condition B, SµG, showed a higher CFU/mL count of 10^9^ corresponding to a OD600 of 1 at week 2 sampling and this was determined to be contamination. Therefore, BR B was removed and only BR A, BR C, and BR D samples T0, Week 1, Week 2, Week 3 and Week 4 were used for further genomic sequencing. Freezer stocks for the three biological replicates (BR A, BR C, and BR D) at each of the three timepoints in the respective growth conditions were streaked onto TA agar and two colonies were selected, cultured overnight at 37 °C in DM2000, and pelleted with supernatant removed. Subsequently, 78 cell pellets (Table 3) were sent on dry ice to Azenta GeneWiz for DNA extraction, library preparation and sequencing. 2 million reads were obtained per genome. Trimmed and quality-controlled reads were compared to the REL606 genome to predict mutations using *breseq* [32]. *breseq* uses Bowtie2 to map reads to a reference sequence [33]. It predicts deletions from regions without mapped reads and other mutations from read alignments, including by using split-read alignments to predict transposon insertions and other structural mutations that create new sequence junctions joining distant parts of the reference sequence [34]. To determine whether any gene amplifications evolved, read-depth coverage plots tiling the REL606 reference genome were generated using the *breseq* BAM2COV utility command and manually screened for regions as small as several hundred bases with increased coverage. Output files were curated by examining evidence for mutations from aligned reads to only include high-quality predictions. An ancestral gene conversion in the ribosomal RNA gene *rrlG* that was present in all samples, including T0, was removed.

**Table 3:** DNAseq sample counts for the long-term simulated microgravity experiment

	BR A	BR C	BR D	Media
T0 (two clones from each BR)	2	2	2	DM25
R (two clones for each BR from Weeks 1,2,3,4)	8	8	8	DM25
S (two clones for each BR from Weeks 1,2,3,4)	8	8	8	DM25
SuG (two clones for each BR from Weeks 1,2,3,4)	8	8	8	DM25
Sample total	26	26	26	78

## Results

### *E. coli* REL606 growth dynamics are not affected under simulated microgravity

**Fig. 2:**
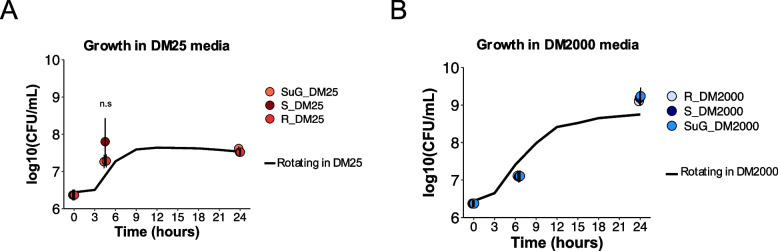
REL606 cell density is not impacted by simulated microgravity. Growth curves were collected for rotating control samples across a 24-h period. Solid line represents growth curves collected for rotating control samples measured at inoculation (0 h) and every 3 h for 24 h in (**A**) DM25 glucose-limited media and (**B**) DM2000, glucose-replete media. Circles represent log10 colony forming units (CFUs) measured at the time of collection for RNAseq for each growth condition in limiting (red circles) and replete (blue circles) glucose media. The DM25 SµG, R and S conditions were sampled as T1 at 4.5 h and as T2 at 24 h. The DM2000 SµG, R and S conditions were sampled as T1 at 6.5 h and as T2 at 24 h. Averages were plotted for the SµG, R and S growth conditions with an *n* = 4. Error bars denote standard deviation. There was no significant difference found among SµG, R and S cultures in the same media

REL606 cell densities were unaffected by SμG compared to rotating or static conditions in both glucose-limited (DM25) and glucose-replete (DM2000) media during the two timepoints at which growth and transcriptomic changes were evaluated. Under SμG, the population retained similar final cell count numbers as expected for DM25 (~ 4 × 10^7^ CFU/mL) and DM2000 (~ 2 × 10^9^ CFU/mL) (Fig. 2A-B). When samples were collected for transcriptome analysis an aliquot was removed to determine cell density at 4.5 and 24 h post inoculation in DM25 samples, and 6.5 and 24 h post inoculation in DM2000 samples. In this study, colony forming units from plating on agar were used to determine cell densities and ensure all cell counts represent live cells. The cell density values of the RNAseq experiment were plotted on a growth curve conducted prior to the RNAseq culturing experiment but under the same environmental conditions. This growth curve was obtained from the rotating control condition in HARVs in DM25 (Fig. 2A) and DM2000 (Fig. 2B) media which were measured at inoculation (0 h) and every 3 h for 24 h. Similar growth curves were not determined for SμG and static samples given the constraints of our experimental set up. Our findings align with a spaceflight experiment using nonmotile *E. coli*, which also reported no change in final cell counts [18]. Overall, these data show that non-motile *E. coli* REL606 reach comparable cell numbers at each of the timepoints that were assayed under simulated microgravity (SμG) in both limited and replete glucose media.

### *E. coli* REL606 activate stress response pathways in simulated microgravity under glucose limitation after 24 h of growth

**Fig. 3:**
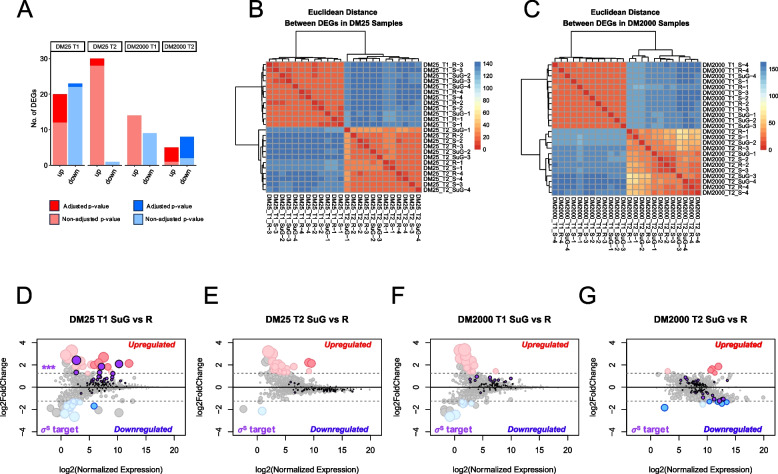
REL606 transcriptomic response to simulated microgravity is altered by nutrient availability. **A** Upregulated and downregulated DEGs per treatment per timepoint are depicted as a bar chart. Dark red and dark blue portions of the bar represent adjusted *p*-values of *p*-value < 0.05 and the lighter red and blue portions of the bar represent non-adjusted *p*-values of *p*-value < 0.05. Euclidean distances visualized through hierarchical clustering of DEGs in (**B**) DM25 samples and (**C**) DM2000 samples. **D-G** MA plots with upregulated at adjusted *p*-value < 0.05 (dark red), non-adjusted *p*-value < 0.05 upregulated (light red), downregulated at adjusted *p*-value < 0.05 (dark blue), and non-adjusted *p*-value < 0.05 downregulated (light blue), condition specific DEGs from each treatment and timepoint comparison. Genes regulated by the general stress response (σ^S^) are shown in purple. The dot size is equivalent to the log2FC. The DEGs with log2FC >  ± 1.25 and *p*-value < 0.05 are depicted. **D** SµG vs R in DM25 at T1, (**E**) SµG vs R in DM25 at T2, **F** SµG vs R in DM2000 at T1, (**G**) SµG vs R in DM2000 at T2. *** = adjusted *p*-value < 0.001 by hypergeometric testing

Bacteria alter gene expression to cope with environmental stressors [35–37]. Therefore, in this study we explored the response to simulated microgravity through transcriptomics. Initial exploratory data analysis identified the time of sampling (T0, T1, or T2) as the variable that drives the largest transcriptomic differences amongst samples (Fig. 3B-C, Supplementary Fig. 1A). Glucose availability did not factor into global gene expression differences at T0 or T1 but accounts for approximately 15% of the variance at T2 (Supplementary Fig. 1A). Importantly, no batch effects were observed between biological replicates (Supplementary Fig. 1B). Each SμG sample was compared to the rotating (R) and static (S) controls independently to identify significantly differentially expressed genes (DEGs) for each condition at T1 and T2. DEG analysis between SμG vs R and SμG vs S yielded similar results with genes associated with various stress pathways and nutrient starvation. When determining which control group to use for downstream analysis (rotating or static) we found that SμG samples associated more closely with static samples as determined by computing Euclidean distances visualized by hierarchical clustering (Supplementary Fig. 1C). DEGs used in the downstream analysis are only those from SμG vs R comparisons because this offers the difference in the rotational axis as a singular change in experimental variable.

Using strict statistical cutoffs of Benjamini–Hochberg adjusted *p*-value < 0.05 and log2FC >  ± 1.25 we found samples at T1 in DM25 produced the largest number of significant DEGs which were mostly upregulated, but overall, only 9 genes were significantly differentially expressed at these parameters (Fig. 3A). Subsequent comparisons produced even fewer DEGs suggesting the transcriptomic signatures captured at these timepoints may not be heavily influenced by short term simulated microgravity exposure. Given the low number of DEGs we relaxed *p*-value cutoffs to non-adjusted *p*-values of *p* < 0.05 and found several more genes with altered expression in each comparison (Supplementary Data 1). Interestingly SμG samples at T2 in low glucose media yielded nearly all upregulated genes, while all other comparisons showed a more even split between up and down genes (Fig. 3A) with non-adjusted *p*-values (*p* < 0.05). These findings further indicate that while gene expression differences are initially distinct between conditions, they become more similar over time, particularly in DM2000 media, where minimal differential expression is observed at T2 (Fig. 3A).

To evaluate the relationships among samples, the DEGs from each of the four comparisons with non-adjusted *p*-values were concatenated to form a list of 105 genes and the expression of each gene was log2 transformed (Supplementary Data 2). These expression profiles were separated by DM25 samples and DM2000 samples and were clustered using Euclidean distances and visualized by hierarchical clustering in a heatmap (Fig. 3B-C). As with the PCA plots in Supplemental Fig. 1A-B the driving variable is time, the samples do however often cluster based on growth condition where 3 of 4 SμG replicates are grouped together at T1 in both media types (Fig. 3B-C). Interestingly, there is little overlap of DEGs amongst the four comparison groups (DM25 T1, T2 and DM2000 T1,T2) with only two genes shared between any of the groups, one of which is a hypothetical protein shared between DM25 T2 and DM2000 T1 and the other *bssR* a biofilm formation regulator shared between DM25 and DM2000 both at T1. *BssR*, showed a log 2FC of -1.68 for DM25 at T1 and a log 2FC of 1.44 for DM2000 at T1. In a previous report, the deletion of *bssR* was demonstrated to increase biofilm formation of *E. coli* K-12 in minimal media supplemented with 0.4% (4 mg/L) glucose [38].

The general stress response is activated when cells are unable to continue dividing normally, and it activates genes to cope with stress [39–41]. Since *E. coli* is known to accumulate rpoS, a transcriptional activator (σ^S^), in times of oxidative stress, osmotic stress, acidic pH, or nutrient deprivation [40], we queried rpoS-regulated genes to profile their transcript levels across conditions. Using a list of σ^S^ regulon members from Weber et al. 2005 [37], the log2 mean expression over log2FC was plotted, which revealed that rpoS targets were upregulated at T1 in SµG samples grown in glucose-limited conditions (Fig. 3D-E). Although some upregulation of σ^S^ targets was also observed at T1 and T2 in glucose-replete SµG samples (Fig. 3F-G), this enrichment of σ^S^ targets was uniquely correlated with the T1 timepoint in glucose-limited samples with an adjusted *p*-value < 4.09e-07 as determined by hypergeometric testing for over-representation of rpoS regulon members. The σ^S^ gene targets which were upregulated in DM25 T1 include *osmB*, an osmotically inducible lipoprotein and *gadE*, an acid resistance transcriptional activator. This finding suggests nutrient deprivation significantly increases stress in the simulated microgravity environment.

### Gene Ontology analysis suggests that simulated microgravity alters expression of genes associated with biofilm formation, cellular aggregation and stress response

**Fig. 4:**
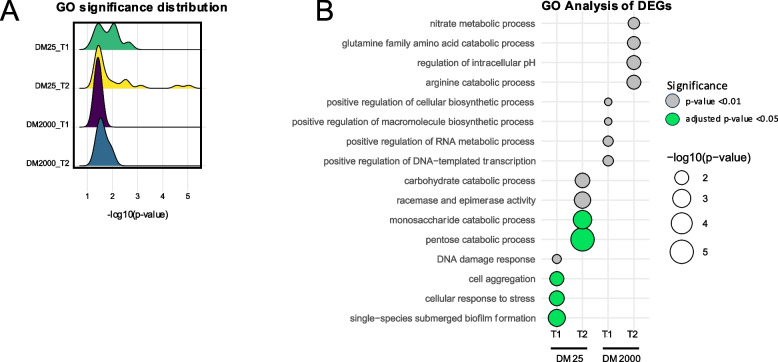
Nutrient availability impacts stress response and metabolic pathways in simulated microgravity. **A** Ridge plot showing the distribution of significance values for enriched GO terms in each group. **B** Dot plot illustrating the significant GO terms associated with the DEGs from each group. Green dots have adjusted *p*-value < 0.05 and gray dots have *p*-value < 0.05

Given the distinct transcriptomic profiles of cells grown in DM25 and DM2000 media, the cellular pathways affected by SµG were investigated. Gene Ontology (GO) analysis with hypergeometric testing with the Benjamini–Hochberg method to adjust the *p*-value was conducted on DEGs (*p* < 0.05) from each comparison. GO categories enriched in DM25 samples had higher significance values than terms associated with DM2000 samples (Fig. 4A). Interestingly, no significant GO categories were shared amongst any of the groups. Like the DEG analysis we found SµG samples at T1 in DM25 had the largest number of significant ontologies with an adjusted *p*-value < 0.05 cutoff. Additionally, at this cutoff we do not find any significantly enriched categories associated with either time point at DM2000, suggesting that potentially no biological pathways are severely hindered by simulated microgravity in higher glucose conditions. rpoS stress response genes are activated by nutrient starvation, and it follows that GO terms categories with an adjusted *p*-value < 0.05 in DM25 samples were associated with biofilm formation and aggregation, stress response, and metabolism. Previous research supports these findings, which showed cellular envelope thickening [42, 43] and enhanced biofilm formation in response to microgravity [44]. When looking at GO categories with *p*-values of *p* < 0.05, we find samples at T1 are enriched for DNA damage and stress response, with a shift to almost exclusively metabolic pathways by T2 (Supplementary Data 3). At the T2 timepoint, 24 h post inoculation, the cells will utilize what nutrients remain [16]. At both time points in DM2000 media the GO terms are largely focused on metabolism and biosynthesis pathways in response to simulated microgravity samples (Fig. 4B). DM2000 samples did not have any categories that met the adjusted *p*-value cutoffs, and these groups also had the fewest number of DEGs suggesting cells may experience less stress when supplied with ample nutrients. These findings indicate that simulated microgravity induces distinct stress responses in *E. coli REL606*, with nutrient availability shaping adaptation strategies—favoring stress response and biofilm formation under nutrient limitation, while metabolic changes are experienced across all groups.

### Long term exposure to simulated microgravity and glucose-limited conditions selects for genotypes with mutations known to affect cell division and membrane permeability

**Fig. 5:**
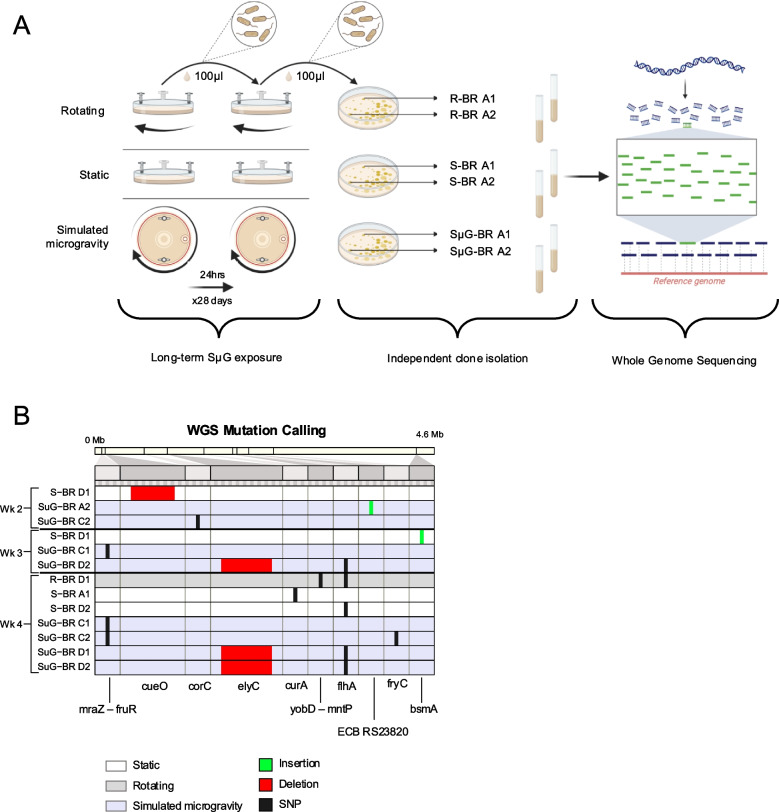
Long-term exposure to simulated microgravity induces genomic alterations. **A** A schematic of the REL606 long-term simulated microgravity experiment conducted in DM25 for the SµG, R, S conditions over 28 days. Four colony isolates of REL606 were inoculated into four separate HARVS (Biological Replicates A, B, C, D) for each condition SµG, R, S and cultured for 28 days. 1% of the culture from each of the 16 HARVs was transferred to a sterile HARV with fresh DM25 media daily. Every 7th day, a sample from each of the 16 HARVs was taken, and a culture stock was made and stored in the -80 °C freezer. The freezer stocks for SµG, R, S of BR A, C and D for weeks 1–4 were each plated on media, and from each, two colonies were isolated for whole genome sequencing. The DNA sequencing reads for each of the two colonies from samples SµG, R, S of BR A, C and D for weeks 1–4 were mapped to the REL606 genome and analyzed using the bowtie2 within the *breseq* bioinformatics tool. Created with BioRender.com (**B**) Mutations found using *breseq* for two colony isolates (1 or 2) from the sequenced biological replicates (A, C, D) for SµG, R, S conditions cultured in DM25 for 28 days and sampled weekly visualized in the context of the whole genome of the REL606 ancestor strain. Samples that did not present mutations are not pictured

Our RNAseq analysis found higher expression of general stress response genes following exposure to SµG (Fig. 3D). Activation of these responses might affect how *E. coli* evolves under simulated microgravity compared to the rotating and static control conditions. To profile what mutations selected over longer-term exposure to simulated microgravity, REL606 cell populations were cultured for 28 consecutive days under SµG, R, and S conditions (Fig. 5A). Daily dilutions and regrowth yielded ~ 6.64 generations per day for ~ 186 total generations of evolution. Figure 5B displays the mutations identified for T0 and weeks 1–4 for the biological replicates (BR) A, C, and D of the long-term culture experiment. Here, two colonies (1, 2) for each BR for each of the sampling events (SµG, R, or S) over four weeks were subjected to whole-genome sequencing (Fig. 5A). None of the three BRs within any treatment had the same mutation patterns (Fig. 5B); however, within BR C and D of the SµG treatment, the same population-specific mutations were observed in multiple colonies beginning at week 3 (Supplementary Data 4). This persistence in the sampled clones suggests that these mutations were highly beneficial and were in genotypes that were outcompeting the ancestor strain [45, 46]. Other mutations that were observed in only one clone at one time point may be in genotypes that never reached very high frequencies within the populations, indicating that they may have been less beneficial or, in rare cases, may have even been neutral or deleterious.

In week 2, the first base substitution was observed in the *corC* gene in SµG BR C2. This gene codes for a magnesium/cobalt transport protein. Also, during week 2 the first insertion upstream of the prophage gene *ECB RS23820* was observed for SµG BR A2 (old locus tag: *ECB_01992*). This insertion increases the number of repeats in a seven-copy four-base repeat in the ancestral genome that has been observed to be hypermutable in other evolution experiments with REL606 [47, 48]. Neither event is observed in clones for the subsequent weeks. During week 2, a deletion appeared in the *cueO* gene in BR D1 in S for one of the two sequenced clones. The *cueO* gene is associated with multicopper oxidase (MCO) involved in copper tolerance under aerobic conditions, and deletion of this gene confers an advantage in iron-limited settings [49]. The 11-base pair deletion was not identified in any clone for any BRs for any treatment in subsequent weeks (Fig. 5B).

In week 3, one colony from BR D1 in the S condition presented an insertion in the *bsmA* gene, but not in subsequent weeks. This gene is named for its role in promoting biofilm stress and motility. One clone of the two BR C colonies sequenced from SµG conditions in week 3 shows a mutation in the *mraZ/fruR* intergenic region that persisted into week 4 for both BR C1 and C2 colonies. The *mraZ/fruR* base substitution may play a role in environmental control of cell division and peptidoglycan synthesis as the MraZ protein is a transcriptional regulator of both processes [50]. In week 4, BR C2 under SµG also shows a SNP event in *fryC*. This gene codes for a putative enzyme IIC component of the phosphoenolpyruvate-dependent sugar phosphotransferase system (PTS), a major carbohydrate active-transport system. The mutation suggests a decrease in metabolism of certain carbohydrates, which researchers also found in the MT-2 effort for the ISS metagenomes.

In week 3, a 13-base pair deletion in the *elyC* gene was identified in the SµG BR D2 sample accompanied by a base substitution in the *flhA* gene. The *elyC* gene mutation pattern persisted into week 4 with the same combination occurring in both SµG BR D1 and D2 samples. In week 4, the same base substitution in the *flhA* gene of the already impaired flagellar machinery of REL606 was also present in the R BR D1 and S BR D2 samples. Given that the *flhA* mutations occurs in S and R samples at week 4 as well, this mutation likely was already present in a subset of cells within the culture used to start all D populations, arising during its initial outgrowth from a single colony. Its parallel rise in frequency in all three conditions suggests that it is beneficial with respect to some shared aspect of the S, R, and SµG treatments, rather than specifically associated with adaptation to simulated microgravity. The deletion in *elyC* which codes for an inner membrane protein shown to alter peptidoglycan biosynthesis rates remained unique to the SµG condition. This *elyC* 13 bp deletion (AGAAGCTGCAGCA) identified in our current study causes a frameshift resulting in a stop codon which truncates the protein. The *elyC* gene also did not evolve mutations in any of the sequenced clones from the Lenski LTEE that maintained the ancestral mutation rate [48]. As the LTEE was conducted in a similar growth medium to these experiments, this observation further supports that the *elyC* mutation may be beneficial in SµG conditions, but we cannot rule out that it may have hitchhiked to high frequency in this population because it occurred in the same genome as a *flhA* mutation. The colony forming units for the REL606 ancestor and an evolved strain with the *elyC* deletion mutation were plated and counted after 24-h growth under SµG, R and S conditions in both DM25 and DM2000 at 25°C and no differences in viability were observed as the conditions produced an average of 4.16 × 10^7^ CFU/mL.

## Discussion

Bacterial cells have evolved to sense their external environment and trigger appropriate internal responses. Selective pressure from the external environment can cause bacterial cells to alter their physiology transiently, through gene expression, or permanently, by mutation to adapt and eventually evolve within a niche. Microbial cells on Earth have evolved over billions of years with gravity as a selective pressure. From space-related microbial research it is known that the secondary effects of microgravity predominate in liquid cultures. These effects include the absence of gravity-dependent cell settlement and a lack of density-driven convection which leads to reduced mass transfer. Thus, microgravity could lead to a more complex environment with spatial structure and cell subpopulations experiencing different microenvironments—and therefore heterogeneous growth phases and stresses—compared to well-mixed or static liquid cultures grown in a terrestrial environment. The response of microbial cells over the long term to the altered physical environment of microgravity is still unknown. For the present study it was hypothesized that prolonged exposure to simulated microgravity would influence gene expression and genome adaptation in *E. coli* REL606.

To test this, first we performed a transcriptomic time course in glucose-limited and replete media in addition to evaluating cell number. Although a more than two times greater cell growth in simulated microgravity reported for prior spaceflight samples was not observed, a transcriptomic signature consistent with other spaceflight [51, 52] and simulated microgravity [53] studies was observed. A previous study investigating the phenotypic effects of simulated microgravity on *E. coli* found that cells experienced cell membrane thickening and decreased membrane transport [43]. A microarray analysis on *E. coli* K12 MG1655 grown in a clinostat to simulate microgravity found changes in the expression of genes associated with membrane transport, transcription regulation, and stress adaptation [1]. Here, as with prior reports, the transcriptomic upregulation of genes associated with metabolism, biofilm formation, and a general stress response were observed across timepoints in both media types during exposure to simulated microgravity. Surprisingly, the DM2000 SµG treatment appeared to overcome some of the stress, yielding the fewest number of DEGs at 24 h with a notable change in the downregulation of *soxS*, a transcriptional activator of oxidative stress genes [54]. However, this is in line with prior spaceflight experiment observations whereby when cells are provided ample carbon, cultures can overcome the signature stresses imparted by the lower convection and mass transfer [16]. Moreover, the expression of genes associated with the general stress response (GSR) regulated by σ^S^, were found to be significantly upregulated for SµG only at T1 in DM25 media (Fig. 3D). A similar phenomenon was observed in a study modeling reduced gravity through clinorotation, where transcriptional signatures like the GSR could be reversed by adding additional nutrients to the reduced gravity samples [55].

It is worth pointing out that many studies have investigated the effects of true and simulated microgravity on various microbial species using different growth platforms. While the results do not perfectly replicate each other, the affected pathways are often the same. Microgravity consistently impacts bacterial metabolism, cell wall/membrane function, and transcription, highlighting these areas as key modes of bacterial adaptation to microgravity stress. Understanding these adaptations has practical implications as *E. coli* can become an opportunistic pathogen under stressful conditions and are regular members of the human gut microbiome. Towards this, previous studies have shown that the gut microbiome is altered in a simulated microgravity bedrest state in humans [56] and that simulated microgravity can impair microbiome function [16]. Interestingly, microbiome samplings had increased population density of beneficial microbes when spaceflight countermeasures were applied [56], and bacterial metabolism showed functional recovery when supplied with excess carbon [16]. These studies and others highlight the importance of understanding the spaceflight adaptive mechanisms of microbes in greater detail to maintain human health in space.

In our current study, it was observed that acute exposure SµG in DM25 induces signatures of stress response, positive regulation of biofilm formation and cellular aggregation. A sustained response in this environment may shape long-term adaptation to simulated microgravity. When explored, in this study, after 21 days of continuous culture, the 13-base pair deletion in *elyC*, associated with increased production of ECA in the gram-negative bacterial cell membrane, was uniquely observed in the simulated microgravity condition. ECA comes in the two outer membrane forms of phosphoglyceride-linked (ECA_PG_) and lipopolysaccharide-linked (ECA_LPS_) and one periplasmic form of ECA (cyclic ECA_CYC_). ECA is involved in maintaining the outer membrane permeability barrier, which excludes toxic molecules, such as antibiotics. A prior transposon mutant library study identified the *elyC* mutant as a clone with increased surface exposure of enterobacterial common antigen (ECA) [57]. Deletion of *elyC* caused continuous production of ECA, due to loss of its wild-type role in regulating the production of ECA peptidoglycan and lipopolysaccharide. *ElyC* mutants in *E. coli* are reported to have slower growth, and are lethal under culture conditions with aeration at 21°C, yet they do not exhibit this response in aerated cultures at 37°C [57] or in low oxygen cultures at 21°C [58]. It is important to point out that our experiments were conducted at 25 °C, the average ambient temperature of the ISS and plant growth habitats on the ISS, whereas the LTEE was conducted at the optimal *E. coli* growth temperature of 37 °C. Additionally, in the longer term SµG treatment, a mutation in the promoter region of the master regulator *mraZ* that controls cell division and peptidoglycan synthesis was also uniquely observed. According to data from the LTEE using REL606, *mraZ* mutations happen relatively early on at ~ 500 generations in this experiment, whereas the *elyC* mutation rarely occurs at all and not before 15,000 generations [48].

The prior long-term simulated microgravity experiment [29] in *E. coli* did not include biological replication or R and S controls. Therefore, it cannot be determined if the observed mutations were because of the simulated microgravity environment or if these would also occur under ambient gravity conditions. A more recent study in *Streptococcus mutans* provided the first example of a well-controlled long-term evolution experiment under simulated microgravity for 100 days of growth [59]. This study did not use independent colonies from the ancestral clone to start each biological replicate to ensure that they did not share any preexisting genetic diversity proclivities, but it did employ replication. We found that mutations in some genes first arose in the simulated microgravity replicates and then later appeared in the corresponding control replicate which was similarly observed in the *S. mutans* study in rich media. Interestingly, both the *S. mutans* and our *E. coli* REL606 study presented mutations in a gene from the phosphoenolpyruvate (PEP)-dependent sugar phosphotransferase system (PTS), a major carbohydrate active-transport system. REL606, in week 4 (28 days) SµG BR C2 showed a SNP event in *fryC*. This gene codes for a putative enzyme IIC component specific for fructose. In the *S. mutans* study, all four replicates presented a mutation in the *ptsH* gene, which encodes for the histidine phosphocarrier (Hpr) protein, and then appeared later in two of the “normal” gravity controls by 63 days of continuous culture. Hpr controls the preferential consumption of glucose over other sugars [60]. Both studies also observed unique mutations specific to the simulated microgravity treatment that were absent in the R “normal” gravity control. However, the simulated microgravity unique mutations were also not observed to occur in more than one of the biological replicates. Much like the historical LTEE, each population of the simulated microgravity treatment appears to be on an independent evolutionary trajectory [61]. It would take additional highly replicated and possibly longer evolution experiments to fully understand how simulated microgravity affects bacterial evolution.

## Conclusion

In this work, our current understanding of the long-term effects of simulated microgravity on the model bacteria, *E. coli* REL606, is detailed. *E. coli* is not only a model organism used in molecular biology and biotechnology to produce biopharmaceuticals but is also a food-borne pathogen; thus, it is important to understand how it behaves in the closed environmental conditions of spaceflight for effective use and monitoring. Transcriptomic analysis of *E. coli* over a 24-h growth period in simulated microgravity revealed that signatures of stress response, positive regulation of biofilm formation and cellular aggregation were impacted by the low-shear modeled stress in low nutrient settings. The results of short-term exposure led us to hypothesize that long-term culturing in low glucose media in the simulated microgravity environment may impose a selective pressure on related pathways. By implementing longer term experimentation, over 28 days of continuous culture, a 13-base pair deletion in *elyC*, associated with increased production of ECA in the gram-negative bacterial cell membrane, was uniquely observed in the simulated microgravity condition. Understanding bacterial responses to long-term spaceflight is crucial and research in relevant environments is essential to support healthy crew and bioregenerative life support systems (BLiSS) for future exploration.

## Supplementary Information

Supplementary Material 1.

Supplementary Material 2.

Supplementary Material 3.

Supplementary Material 4.

Supplementary Material 5.

## Data Availability

Sequencing data is available at NASA’s Genelab under the project name OSD-728: E. coli REL606. RNAseq count data is available under GEO Submission (GSE284563). (https://www.ncbi.nlm.nih.gov/geo/query/acc.cgi?acc=GSE284563).

## Abbreviations

SµG: Simulated microgravity culture
R: Rotating culture
S: Static culture
ISS: International Space Station
BLiSS: Bioregenerative life support systems
DEGs: Differentially expressed genes
MT: Microbial tracking
MOA: Mechanism of action
BR: Biological replicate
GO: Gene ontology
LTEE: Long-term evolution experiment
