# Supplementary material for: Simulated microgravity triggers a membrane adaptation to stress in E. coli REL606
Source: BMC Microbiol. 2025 Jun 9;25:362. doi: 10.1186/s12866-025-04064-7 (PMC12147273; doi:10.1186/s12866-025-04064-7)
Supplement: Supplementary file 1 — Supplementary Material 1. [file 12866_2025_4064_MOESM1_ESM.pdf]

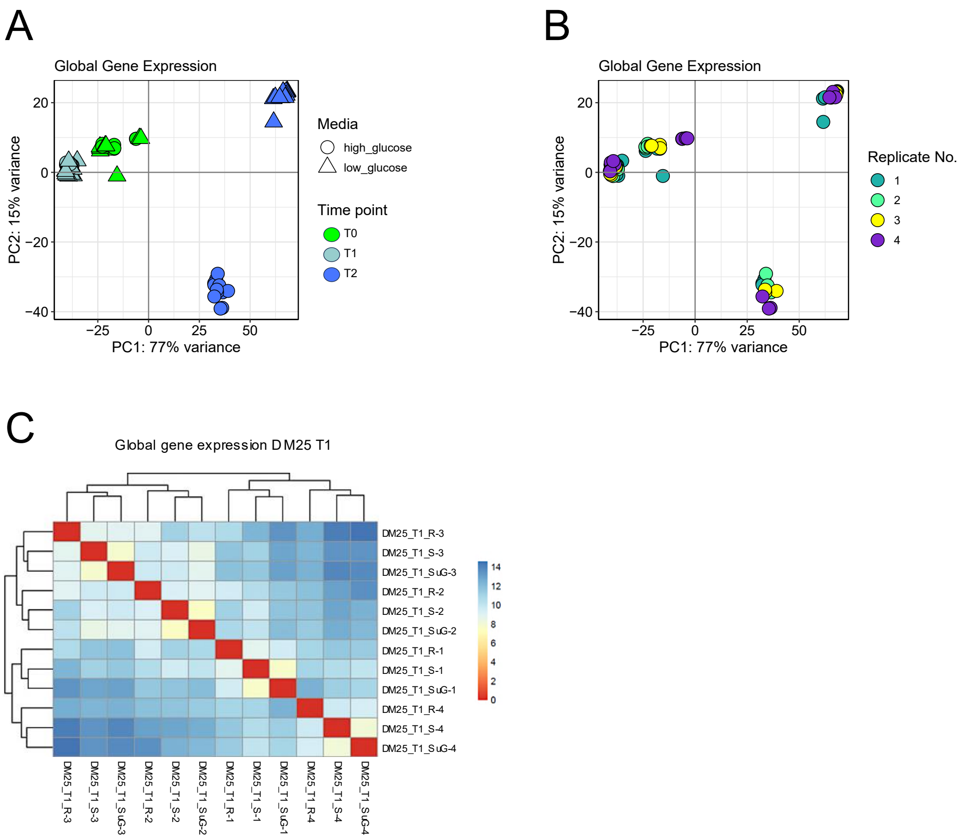

FIGURE S1 | Breakdown and filtering of *E. coli* REL606 transcriptomic data. (A-B) Principal Component Analysis (PCA) of global gene expression annotated by **(A)** timepoint and media type and **(B)** biological replicate group. **(C)** Global gene expression in DM25 samples at T2 with hierarchical clustering across groups.

Supplementary Data 1 | DEGs results from SμG vs R comparisons. (Sheets 1-4) DEG analysis of SμG versus Rotating control in DM25 and DM2000 at T1 and T2. Bolded text represents data with adjusted pvalue < 0.05 while not bold is p <0.05 not adjusted. Protein Accession, and Protein Product subset from REL606 GTF reference genome file. Empty cells equal no available data.

Supplementary Data 2 | Normalized counts of DEGs used for PCA analysis. Log2 normalized counts per million (CPM) for all 102 DEGs pooled across comparisons in DM25 samples and DM2000 samples.

Supplementary Data 3 | Gene Ontology analysis of DEGs from all comparisons. Significant gene ontology (GO) terms, ID, pvalue, and gene ratio for DEGs. Description column represents the specific category called, also identified by Term.ID column.

Supplementary Data 4 | Mutation call on Whole Genome Sequencing after long-term exposure to SμG using Breseq. Long ID represents week sample was collected. Growth condition (S, R, or SμG). Base location identifies the location in the genome where the mutation was found. Mutation type (del = deletion, ins = insertion, snp = single nucleotide polymorphism). Delta bases denotes the number of based lost if mutation was a deletion. Old annotation is the gene symbol from EcoCyc. Gene symbol REL606 is the corresponding annotation using the most recent REL606 assembly (NC_012967). Locus ID corresponds to gene symbol also using the most recent REL606 assembly. Variant details NC_012967.1 details nucleotide and position specific of each mutation. Fasta gene number indicates the number of a gene's location within Fasta file. Basepair substitutions (synonymous or non-synonymous) denotes effect of specific mutation.
